# Prolongation of Pregnancy by Rest in the Last Month

**Published:** 1914-05

**Authors:** 


Prolongation of Pregnancy by Rest in the Last Month, Grenier, *Jour. de Med. de Bordeaux*, October 28, 1913 reports an increase of average from 265 to 286 days.

